# Outdoor fitness equipment in parks: a qualitative study from older adults’ perceptions

**DOI:** 10.1186/1471-2458-13-1216

**Published:** 2013-12-21

**Authors:** Hsueh-wen Chow

**Affiliations:** 1 Graduate Institute of Physical Education, Health & Leisure Studies, National Cheng Kung University, No1, University Road, East District, Tainan City 70101, Taiwan

**Keywords:** Asians, Physical activity, Older adults, Neighborhood park

## Abstract

**Background:**

The growing amount of outdoor fitness equipment (OFE) placed in parks in many countries has the intent of encouraging physical activity among aging populations. However, little investigated aspects are the perceptions and experiences of older adults regarding the use of these facilities. Hence, this study seeks senior OFE users’ opinions to understand the exact nature of use of these facilities, the perceived health benefits achieved, and equipment’s improvements that would further encourage use.

**Methods:**

The study conducts semi-structured interviews with 55 senior OFE users at two parks in Taiwan.

**Results:**

Study results suggest that although OFE use is not the main purpose for which seniors visit parks, most seniors perceive the benefit of using OFE in terms of both physical and psychological health, as well as social connection. Respondents also raised issues related to safety, maintenance, and management of OFE.

**Conclusions:**

OFE appears to meet the health needs of seniors, but improved management is necessary to ensure safety. Further research would reveal the actual behavior involving OFE use and use’s relationship to the physical activity of seniors.

## Background

In response to worldwide aging populations [1,2], many efforts have attempted to improve the health and quality of life of older adults [3,4] such as “active aging” promoted by the World Health Origination to increase physical activity among seniors [5]. A plethora of research has proven that regular physical activity contributes both positive and preventive factors for maintaining health in older adults [4] including physiology [6-10], psychology [11] and cognitive benefits [12,13]. Despite scientific evidence, the number of older adults participating regularly in physical activities remains low in many countries [14-16].

Recently, the socio-ecological model has attempted to explain behavior related to physical-activity among general populations. Marcus and Forsyth [17] indicated that environmental designs and policies are much more effective than programs in terms of sustainability and reaching populations to influence the public’s level of physical activity. Therefore, knowing how to improve the environment to encourage seniors to participate in physical activities is essential.

Since parks’ locations, usually in nearby neighborhoods, are either free or low-cost to visitors, and are accessible, recognition of parks’ importance as settings for physical activities has increased among those with interest for encouraging health [18-23]. Parks, widely used by a surrounding community, are particular favorites of older adults who have limited mobility [24].

Factors influencing older adults’ visits to parks include accessibility [19], perceptions of safety [25], presence of facilities [26], park size [27], things to watch [26], events to attend [25], and maintenance [25,26]. However, studies also show that despite frequently visiting parks, older adults, more so than younger visitors, usually engage in more sedentary activities, such as chatting with friends, sitting and resting, or watching others [28,29]. Kaczynski [27] observed 33 parks in Canada and found that use of parks for physical activities is more likely at those with more features/facilities, while factors such as size, distance, and amenities were not significantly predictive. Few studies, however, have investigated how specific features in a park affect seniors’ physical activity.

In many Asian countries, including China and South Korea, outdoor fitness equipment (OFE) in parks has become very popular. According to Chow [30], more than half of the parks in Taipei city and Tainan city have installed OFE. Although the use of such equipment is not high on other continents, countries such as Spain [31,32], Portugal [33], and the United States [34] reported rapid increases in the amount of this type of equipment in parks and in the market for outdoor fitness machines.

With the appearance and growth of OFE, whose designs and shapes are similar to those found in gyms, parks with such equipment have gained specific identifications, such as Fitness Zones [34], senior playgrounds [33], or geriatric parks [31,32]. The latter two names indicate the equipment’s designs have specific application for older people.

Despite the availability of OFE for seniors, little research has considered older adults’ perceptions of, and experiences with, using this equipment. Whether or not OFE meets the needs of older adults, the details surrounding its use remain unclear. As Cohen [25] asserted, more research needs to investigate which characteristics and conditions of parks promote the greatest physical activity and utilization. Therefore, this study interviewed seniors who are current users of OFE in an effort to understand the exact nature of use of these facilities, the perceived health benefits achieved, and equipment’s improvements that would encourage use. This study can assist discovering seniors’ perception of, and needs for, OFE, thereby providing insight contributing to the planning and design of parks to improve park-based physical activity among older adults.

## Methods

Since little research, which on seniors’ perceptions of using OFE in parks exists, qualitative research is appropriate for eliciting useful, in-depth information leading to understanding individual attitudes, beliefs, and perceptions within a culture [35]. This study obtained approval from the Institutional Review Board (IRB) of National Cheng Kung University Hospital (ER-99-375) for the research and its specific protocol. The following subsections detail descriptions of the methodology for conducting the qualitative approach.

### Data collection

#### Study area

**Figure 1:**
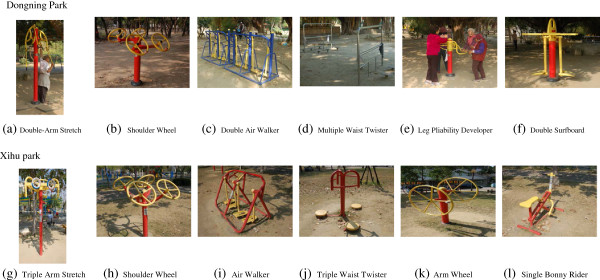
Photos of each type of outdoor fitness equipment in two parks.

The qualitative fieldwork began with several preliminary on-site periods of observation at several parks in the five districts in Tainan city, with the intent of exploring the patterns of OFE used in the parks. Based on the list of parks provided by Tainan city government and an earlier field observational project in each park during January 2012 to May 2012, 86 parks of 132 (65%) in Tainan had installed OFE [30]. To best represent a wider spectrum of parks’ attributes in terms of sizes, locations, neighborhood demographics (i.e. population and proportion of older adults), and socioeconomic status (i.e. poverty rate) led to selection of two parks: Dongning Park and Xihu park. Table 1 summarizes these parks’ attributes, neighborhood characteristics, and older population’s demographics. A comparison of two parks reveals that Dongning Park is a larger facility near a low density of an older population (17.6%) and a neighborhood of relatively high socioeconomic status, and Xihu park, which is small, in an area with a high density of aging population (28.1%) and a relatively disadvantaged socioeconomic status. The two parks which were the sites for the interviews, each had six pieces of OFE equipment (Figure 1). Since weather greatly influences outdoor behavior, climate information at the study sites is: The mean temperature in Tainan city, is 24.3 Celsius, with an average of 87.4 rainy days per year (number of days with precipitation > =0.1 mm) and a mean relative humidity of 77.2% [36].

**Table 1:** Profiles of parks for recruiting OFE users

	**Dongning Park**	**Xihu Park**
Park size (acres)	5.40	0.84
Locations	East District	Central-west District
Neighborhood population^1^	100,226	40,180
Rate of poverty^2^	1.21	2.55
Number of adults >65 yrs	17,600	11,280
% of older population (>65 yrs)	17.60	28.10

Data source: Tainan City Government Website (http://www.tainan.gov.tw/tainan/).

Note:

^1^Neighborhood population refers to the total population in each district.

^2^The percentage of low income households (number of low income households in each district divided by the total number of low income households in the city represents the rate of poverty). The city’s average percentage was 1.48% in 2011.

#### Recruitment of participants

Adoption of purposive sampling directed selection of participants who could provide information relevant to the research’s focus [37] and consequently, at the two parks chosen for the case studies, approached seniors (>50 years old) who are OFE users, who could provide valuable insights into their experiences from using these facilities, and who indicated a willingness to be interviewed. The inclusion criteria limited age to 50 years and above, since that standard has wide acceptance for identifying older adults in established literature (in both survey-based and interventional studies) and in national guidelines that promote physical activity and surveillance for public health [11,38-41]. The 50 and older criterion is also prevalent in environmental gerontology studies that investigate the relationship between built-environment and older adults’ behavior [41,42].

#### Study procedures

Prior to conducting the field survey, the assistants received training in protocols for conducting interviews with senior citizens and obtaining useful results, note-taking methods, and techniques for eliciting expansive responses from the participants. The interviewers received immediate supervisory support upon encountering any difficulties during the interviewing sessions.

Willing participants received initial briefings by either the researcher or the trained interviewers as to the purpose of the study, notification that the interviews would be audio-recorded, and all data, with respect to both demographic information and behavioral information would remain confidential. Participants signed informed-consent forms approved by the Institutional Review Board (IRB) before an interview. The interviews occurred at the outdoor fitness equipment areas in the parks, and each interview lasted between 20 and 45 minutes. As a token of appreciate for the time and effort needed to share experiences, participants received a gift set (value $ 4 USD) upon completion of the interviews. Notes from observations, recorded on–site, accumulated richer information from any observations or interactions with the participants. After the interviews, a peer debriefing double checked the content and accuracy of interviews and field notes.

### Research instrument

Since the purpose of this study is to explore seniors’ perceptions of OFE and their experiences using these facilities, development of a semi-structured interview guide pursued the same basic lines of inquiry with each respondent. The guide used in the interview process aligns with the main research questions. In addition to the guide in text-form, color photos of individual machines, accompanied by its proper name, acted as prompts during interviews, assisted participants’ accurately identifying the equipment during the interview, and facilitated the process of transcribing the interview. The semi-structured interview guide and photos appear in Table 2 and Figure 1, respectively.

**Table 2:** Semi-structured interview guide

	**Questions**
1	• How often do you visit the park?
2	• What did you usually do when visiting the park?
3	• Why do you want to use these outdoor fitness facilities in the park?
4	• What are the facilities you like to use most?
5	• When do you come to use these facilities during the day (morning, afternoon?)
6	• Do you usually use these facilities alone or with someone else?
7	• How long do you use these facilities typically?
8	• In your opinion, what is the benefit of using these facilities?
9	• Do you encounter any problems or injury in using these facilities?
10	• Do you think these facilities fit seniors’ needs?
11	• Do you have any recommendation for these facilities such as the types? Locations? Or areas?
12	• In terms of your physical activity level, do you perceive any changes of physical activity level after the installation of the fitness equipment? How long have you used this equipment?
13	• Any other suggestions or ideas you would like to express?

### Data analysis

Several reviews of the transcripts and rechecking with the interviewers assured accuracy. Further analysis occurred after importing the texts into qualitative data management software, Nvivo 8 (QSR International Pty. Ltd., Melbourne, Australia). Then, an interpretive process investigated similarities and differences among individuals. A continuing comparison and contrasting of themes within and among individuals, guided by Glaser and Strauss [43], was adopted, and “axial coding” [44], examined the connections among codes, to determine the relationship among links. The actual coding framework was more open-ended and underwent revision if a previously overlooked element became apparent. The field notes, which constitute information from observations of senior participants’ use of the OFE and their social interactions onsite, served to supplement the interview data to increase authenticity and trustworthiness of the findings. The analytic processes, initially independently identified by the researcher and interviewers and cross-validated, produced a consensus during the research’s planning meetings. When new themes emerged with new interview data, the existing codebook gained additional codes. Conducting 55 interviews achieved thematic saturation: the point at which no new codes would likely emerge from additional research participants. The finalized codebook contained detailed descriptions of the themes and their patterns. Three participants agreed to verify the descriptions and coding well represented their experiences. Based on the consensus for the codebook, the researcher and the graduate student independently coded all of the interviews’ source material. Coding comparative analysis with Nvivo software assessed the degree of agreement between the two coders. Percentage of agreement, percentage of disagreement, or Kappa Value illustrated the results of comparison. As in Nvivo, the percentage of agreement/disagreement tends to be higher since the software calculates both un-coded and coded text. The Kappa value, which considers “the respective length, and duration or size of each source being considered for agreement” [45], is a better representative. The Kappa value for the two coders in this project was 0.82, which represents a good indicator for a high degree of agreement, based on the criteria of Landis and Koch [46].

Finally, since the original transcripts are in Chinese, the quotes presented are the result of translations into English and then back-translated by two faculty members, who are fluent in both English and Chinese. This verification determined whether or not the original and translated quotes are consistent in terms of content and language.

## Results

The following sections present the demographic characteristics of the respondents and describe themes and subthemes found in the interviews and field notes. The organization of the themes with quotes best faithfully captures common topics in the seniors’ words and clearly addresses the aims of the study. To maintain anonymity, the names of the parks and the order of interview substitute for the names of the participants. For example, the first participants interviewed in Xihu Park received the pseudonym of X-1. The participants’ genders and ages appear with each selected quote.

### Characteristics of participants

A total of 55 seniors (27 males and 28 females) provided interviews for this study. Seventeen of them were between age 50 and 60, 13 ranged between 61 and 70, 20 between 71 and 80, and two participants were above age 81 with an oldest, 97 years old. Interviews for 22 of the 55 participants occurred in Dongning Park and 33 in Xihu Park. Most of the respondents came to the parks alone. Of the 55 seniors interviewed, 78% indicated that they exercise in the park daily, 13% approximately three times per week, and 9% twice per week. Most of the respondents visit the park in the early morning, for one to two hours.

### Use: additional park features for seniors’ activities

Most of the seniors interviewed do not visit the park specifically to use the outdoor fitness equipment; instead, they participate in group exercises or walk; using OFE represents only a supplementary activity. The older adults perceive various pieces of OFE as the park’s additional features that are fun to use. Therefore, some older adults see OFE as a “playground” rather than a resource for “exercise” equipment.

I came to the park for group exercise, and I am heading back home soon to do the laundry. But before I go, I’m going to play on the equipment a little while. (X-25, female, 82 yrs. old).

Based on the participants’ responses, the duration of equipment use varied between 5 to10 minutes and 1 hour. One respondent indicated that seniors, in general, do not use OFE vigorously.

As we are old, our exercise does not need to be that rigorous. (X-25, female, 82 yrs. old).

However, even moderate use of the OFE causes older adults to sweat; as one respondent said:

Although we don’t use this equipment very rigorously, using it does cause us to sweat, which is good (D-03, female, 69 yrs. old).

Most interviewees reported that they used various pieces of OFE; nevertheless, the most popular among the respondents was the arm stretch, as most of users reported shoulder problems. Field notes recorded observation of several wheelchair-bound older adults accompanied by caregivers in the OFE area; however, while the caregivers used the OFE, the physically compromised older adults either did not exercise at all or did a few arm-stretch exercises.

### Using OFE for health improvement and as pastime

For most of the older adults interviewed, the main purpose of using OFE is to exercise and improve health. Some respondents mentioned that parks’ OFE is an ideal location for its enjoyable natural environment. As one female respondent reported:

We have had a stationary bike in our house for a long time, but we rarely use it. We like to come to the park to breathe fresh air and use this equipment because it feels more like exercising while coming out (D-03, female, 69 yrs. old).

Some of the respondents viewed OFE as an opportunity to pass time and relieve boredom.

You know…sometimes we just want to kill time in the park, and if we keep our legs moving while we chat with others, we feel good about ourselves because we are exercising (X-27, female, 57 yrs. old).

### Benefits of using the equipment for stretching, rehabilitation and to improve mood

Although most of the respondents were unable to identify specifically the types of physiological benefits gained from using the OFE, they agreed that the equipment did enhance health. As one female senior mentioned:

I don’t know exactly if my body has improved or not, but at least, I am exercising and that will lead to better health (D-09, female, 61 yrs. old).

Several of the respondents indicated use of equipment to stretch or as a station for massage to decrease muscle soreness or stiffness. This is especially the case for those suffering from frozen shoulder symptoms, lower back pain, and osteophytes. One female respondent said:

I have frozen shoulder problems, so I came to the park to do some arm stretches, and then, I came frequently to do the pull. Now, I feel that my shoulder is getting better and becoming more relaxed. (X-01, female, 60 yrs. old).

Another female, who previously participated in folk-dance activities in the park, reported that a car accident several years ago resulted in a six-month inability to walk during the rehabilitation. Since her knees could not support her body for extended walking, she has been using the equipment in the park for almost 10 years to help regain lower-limb strength (D-19, female, 75 yrs. old). Another male respondent indicated that he has osteophytes and purposely visits the park to use the equipment for rehabilitation.

I have seen neurosurgeons, who told me that medication is useless. I have to have surgery and then go through rehabilitation. I did not follow his suggestion; instead, I came to use the equipment and found them to be effective (X-24, male, 58 yrs. old).

Besides physical benefits, many of the respondents thought that the equipment improved their psychological well-being.

You will feel happier after using the equipment. It is good. (D-21, male, 84 yrs. old).

### Social interaction: developed new friendships from frequent visits

Although most of the respondents visit the park alone, they mentioned the equipment area as a social setting:

I usually come here alone and my husband would join me later, but it doesn’t matter because I come here frequently, and I know most the people in the area; it is fun just to be here (X-11, female, 77 yrs. old).

You come here frequently and you become familiar with the other people here, then, you become friends (X-07, female, 60 yrs. old).

### Availability: more OFE needed but space is a concern

Although some respondents think that the amount of OFE in parks is adequate, others think more, during peak hours of use, especially in the early morning or late afternoon, would be advantageous.

I have to take turns to use this equipment, and it is embarrassing to ask those using the equipment to give others a turn. Some people only sit on the equipment to rest, rather than exercise (D-13, male, 70 yrs. old).

Although some identified a need for more or different types of OFE, they recognized the availability of limited space:

We do want to have more equipment, but there is not enough space in the park. One must be careful not to place the equipment too close to each other (X-11, female, 77 yrs. old).

### Safety concerns: not a critical problem, but not suitable for children

In general, many older adults do not perceive any safety issues with use of OFE; nevertheless, a few did perceive some risks.

There are no stoppers in most of the equipment; for example, the wheels continue turning without stopping, and that is dangerous (X-18, male, 72 yrs. old).

Some mentioned that falling as a concern; others expressed the sentiment that use of OFE by children seems to be very dangerous.

### Maintenance and management: improvement needed

Equipment maintenance and placement have become serious problems. The respondents mentioned that the equipment needs constant maintenance because of rust (X-21, female, 52 yrs. old) and needs lubrication (D-04, male, 97 yrs. old). Several pieces of the equipment are on uneven ground, which does not provide stability, and causes water to accumulate after rain. In addition, respondents suggested installing the equipment under trees to provide shade to avoid sunburn and relief from summer heat.

Several respondents also mentioned that equipment handles and seats should be softer for more comfort. One male respondent said:

The bonny rider is too hard to sit on, and it will increase friction during exercise (X-18, male, 72 yrs. old).

### Operation of equipment: most seniors have developed an individualized style

Since no instructions for use accompany the OFE, most seniors, do not have clear ideas for using the machines and their functions.

…but we older adults don’t know how to use these equipment (D-16, male, 77 yrs. old).

Hence, many report having developed their own ways or mimic others’ operation of the equipment. One participant said,

It doesn’t matter how you operate it as long as using it provides benefits (D-12, female, 75 yrs. old).

## Discussion

### Summary of study’s findings

Parks in Asia, Europe, and North America have installed significant numbers of OFE, with the claim of meeting the needs of rising aging populations. However, little research has investigated the perspectives of older people in terms of whether or not this equipment accommodates their specific needs or identifies this population’s experiences using this equipment. This study analyzes responses gathered from semi-structured interviews with older people who use OFE and presents the responses categorized according to detailed themes acquired through quotes. Two main themes classify the responses for identifying older people’s perceptions of OFE and their experiences with use.

#### Older adult’s perceptions of OFE

The themes from the interviews show that most older adults use OFE to supplement main activities: group exercise and walking in the park. They also use the OFE for enjoyment, to improve health, and as a means to socialize with others. Many of the respondents perceived symptoms of declining physical functions, such as joint mobility or injuries needing rehabilitation. Besides physical benefits of using OFE, such as increased motion range, improved cardiovascular function, and decreased muscle soreness, respondents also cited psychological and social benefits of using OFE. For example, they expressed that their moods improved, and they enjoyed interacting with other people while using OFE.

These findings support the ideas of Aparicio [32], who stated that using OFE involves all aspects of the human body, including balance, coordination, strength, elasticity, mobility, and agility. In addition, OFE helps treat specific injuries or provides rehabilitation previously available only in gyms and clinics. In addition, OFE also facilitated social interactions among senior OFE users.

Kaczynski et al., [47] suggested that many park visitors, especially adults, remain sedentary during their visits [48]. This suggests that the provision of OFE provides opportunities to increase seniors’ physical activity, which can improve health through operating the equipment while visiting the park. In particular, older adults acknowledged that operating OFE with low intensity could have substantial health benefits and rehabilitative effects; however, the exact energy expenditure and intensity measurements of operating the OFE are unclear. Cohen et al. [34] adopted the System for Observing Play and Recreation in Communities (SOPARC) [49] and estimated OFE users’ activity levels, measured by METs^a^, increased from 15% and 8% from a baseline to 1st and 2nd follow-ups, respectively. These findings are consistent with research that indicates providing more settings for activity or existence of facilities in parks positively relate to park use [26,50,51]. Moreover, OFE also offers a setting for many older adults residing in urban areas with a place to meet and talk with others, creating an ideal situation for older adults to form social ties with neighbors, and consequently, a beneficial sense of social integration for well-being [52].

#### Older adults’ experiences and suggestions for OFE

Although several older adults use all available OFE in the parks, the most popular OFE, according to participants, was the arm stretch machine, as many older adults reported having shoulder problems. OFE designed to increase flexibility (i.e., activities designed to preserve or extend range of motion [ROM] of a joint) appears to be a favorite of many seniors. Research has demonstrated that preserving seniors’ ROM can affect physical functions in particular by assisting independent living [53,54]. Thus, the OFE in the present study appears to meet the needs of seniors’ health concerns.

Most seniors did not express serious safety concerns regarding OFE, but they voiced concern for maintenance and placement of OFE. Although OFE designs include water-resistant materials attention for outdoor environment, nevertheless, requires regular maintenance, especially after long-term installation. Local weather is a consideration since humidity in Taiwan is, overall, high and the OFE can quickly rust if not regularly maintained. Consideration of the role of weather conditions, such as the length of mild-weather periods during which older people tend to be outdoors should also be an aspect when planning OFE installations in other regions.

Since sufficient instructional labels, indicating appropriate users, age-specific restrictions, and instructions are absent, participants in the current study related development of individualized methods for operating the OFE, in part by observing others. Despite the current data’s documenting no injuries, the media has recently reported several accidents using OFE. For example, many children use OFE without adult supervision and experience injurious falls. In fact, Cohen et al. [34] found that, in California, other than adults, children account for about one-fifth of total fitness zone users. Similarly, in Portugal, researchers found that more than 44% of OFE users are children [33]. Therefore, an urgent need is to establish safety requirements for OFE, similar to policies for children’s playgrounds.

Regarding safety for adults, Apricio [32] proposed recommendations for clearance between each OFE device. Since an increasing number of OFE installations are appearing in Europe and the U.S. [51,55], and in spite of these installations offering opportunities for more convenient and no-cost options for the public to be active, safety concerns should be the top priority to avoid injuries.

### Study’s contributions and limitations

A large number of studies investigated the relationship between parks and physical activity among older people. Although parks in Asian countries have a considerable number of OFE and substantial growth of installations is apparent in European countries and in the United States [34], to the best current knowledge, no study has examined OFE specifically from the perspective of seniors or through in-depth interviews. Since this study exclusively considers Taiwan, whose parks have had OFE for a longer period compared to other non-Asian countries, most of the participants reported having used OFE for two to ten years. Hence, the participants were able to address the limitation raised by Cohen [34] regarding sustainability, and these respondents provided extensive information and recommendations than older people in countries in which OFE is fairly recent.

While this study uses criteria and methodology that enhances the scientific rigor of the project, several inherent limitations, characteristics of qualitative approaches, exist, and caution is appropriate when interpreting results. First, as the purpose of this study was to understand the experiences of using OFE among older adults, the study employed a purposive sampling technique, in a deliberative and non-random approach [56]. Thus, this method might be subject to sampling and non-sampling error. Second, since this study represents one limited geographical urban area, involving two case-study parks in Taiwan, the findings cannot be generalized for other regions because environmental factors such as weather, location, and accessibility to OFE in parks greatly influence outdoor behavior. For example, uncomfortable temperatures or bad weather (rain, snow, wind, or heat) can decrease the desire among older adults to leave home. Finally, personal (physical, psychological, and social) and societal factors within a culture shape physical activity among older adults. For example, unlike older adults in other countries, whose participation in physical activities decreases with age [16,57], research conducted by Lin, Wen and Wai [58] found that adults over 45 years show a higher level of regular participation in physical activity than those in the 25 to 44 year demographic in Taiwan.

## Conclusion

Overall, the findings in this study are consistent with those of other studies of physical activities in parks among older adults [21,59]. According to the data in this study, seniors believe that using OFE in parks contributes to their perceptions of promoting health by providing not only physical but also social and psychological benefits. The information in this study also has implications for OFE stakeholders, such as manufacturers, urban planning professionals, park and recreation administrations, and local authorities for enhancing equipment designs and safety regulations to ensure that OFE installations maximize benefits and minimize drawbacks. For example, age-restriction signs to prevent children from being injured in OFE areas are necessary. Continued maintenance is also a requirement for OFE sustainability.

In addition, the OFE in this study represent only eight types (See Figure 1) which is not a comprehensive sample of OFE options available in the market, but rather reflective of the most popular facilities in neighborhood parks in Taiwan. Since many types of OFE are currently available, future studies could investigate these other options in relation to the perceptions and experiences of seniors. Furthermore, this study interviewed only seniors residing in communities with relatively independent OFE users. Senior non-OFE users, those with negative perceptions or experiences, or older adults with limited mobility, who never use or ceased use OFE may provide better insight in terms of barriers to use or other specific needs.

Since little investigation related to OFE appears in the literature, more research is necessary to consider, for example, frequency, duration, intensity, and expenditure of energy of older adults’ using OFE, the relationship between OFE use and the status of older adults’ health, the significance and impact of physical activities involving OFE on seniors’ health, and functional performance after using OFE. A comparison of the differences between OFE and traditional gym facilities in terms of an individual’s functional performance would add a valuable insight. The aspects of design for both OFE in terms of equipment and supporting environment (e.g., OFE along walking trails or within a confined area) need investigation. More study in collaboration with multiple disciplines and various regions would expand the useful results of investigate into the impact of OFE in promoting active aging among seniors.

Although a series issues remain for examination, the present study explored experiences of using OFE among older people. These findings may be used to assist planning and designing parks as well as research to improve park-based physical activities for older adults.

## Endnote

^a^METs or metabolic equivalents, is a common measure in sports’ science indicating the intensity of an activity. One MET represents an individual’ energy expended while at rest, and two METs are twice the energy expended while at rest, etc.

## Competing interests

The author declares that she has no competing interests.

## Authors’ contributions

HWC conceived the design, analyzed the data and drafted the manuscript.

## Pre-publication history

The pre-publication history for this paper can be accessed here:

http://www.biomedcentral.com/1471-2458/13/1216/prepub
